# A Study on Sustainable Concrete with Partial Substitution of Cement with Red Mud: A Review

**DOI:** 10.3390/ma15217761

**Published:** 2022-11-03

**Authors:** Hisham Jahangir Qureshi, Jawad Ahmad, Ali Majdi, Muhammad Umair Saleem, Abdulrahman Fahad Al Fuhaid, Md Arifuzzaman

**Affiliations:** 1 Department of Civil and Environmental Engineering, College of Engineering, King Faisal University, Al-Ahsa 31982, Saudi Arabia; 2 Department of Civil Engineering, Swedish College of Engineering, Wah Cantt 47070, Pakistan; 3 Department of Building and Construction Techniques Engineering, Al-Mustaqbal University College, Hillah 51001, Iraq; 4 Service Stream Limited Co., Chatswood, NSW 2067, Australia

**Keywords:** red mud, eco-friendly concrete, durability, slump, mechanical strength

## Abstract

Every year, millions of tons of red mud (RDM) are created across the globe. Its storage is a major environmental issue due to its high basicity and tendency for leaching. This material is often kept in dams, necessitating previous attention to the disposal location, as well as monitoring and maintenance during its useful life. As a result, it is critical to develop an industrial solution capable of consuming large quantities of this substance. Many academics have worked for decades to create different cost-effective methods for using RMD. One of the most cost-effective methods is to use RMD in cement manufacture, which is also an effective approach for large-scale RMD recycling. This article gives an overview of the use of RMD in concrete manufacturing. Other researchers’ backgrounds were considered and examined based on fresh characteristics, mechanical properties, durability, microstructure analysis, and environmental impact analysis. The results show that RMD enhanced the mechanical properties and durability of concrete while reducing its fluidity. Furthermore, by integrating 25% of RDM, the environmental consequences of cumulative energy demand (CED), global warming potential (GWP), and major criteria air pollutants (CO, NO_X_, Pb, and SO_2_) were minimized. In addition, the review assesses future researcher guidelines for concrete with RDM to improve performance.

## 1. Introduction

To make constant strides toward sustainable growth, a massive revolution in the cement and concrete sector is required to decrease environmental pollution, particularly carbon dioxide [1]. Concrete manufacturing has increased in recent decades, and it is currently one of the top concerns of scholars who are interested in sustainable development [2,3,4,5]. The utilization of waste materials in concrete manufacturing has been shown to minimize natural resource use [6,7,8,9]. Construction uses more raw materials and energy than any other economic activity on the planet today. Concrete is a common construction material used all over the globe. Cement, being a fundamental component of concrete, is produced via an energy-intensive process. Cement manufacturing produces a lot of greenhouse gas emissions, which contribute to global warming [5,10]. The cement factories are the second-largest industrial carbon dioxide emitter, accounting for 5 to 7% of total CO_2_ emissions [11,12]. The use of various waste materials as cement substitutes has been researched for CO_2_ reduction [13,14,15]. In contemporary civilizations, using garbage as a secondary material in the building sector is a cost-effective and environmentally friendly means of disposing of waste [16,17]. Sustainability in the construction industry has become crucial, and numerous solutions have emerged to lessen the environmental effect of present building operations [18,19]. Rising energy supply costs, decreasing CO_2_ ejections, and the delivery of unrefined, low-quality ingredients all pose risks to the cement business [20]. As a result, alternative suppliers must be sought instead of cement. According to research, concrete created from waste materials such as plastic waste is one of the waste disposal alternatives [21]. Furthermore, in this century, most researchers are concentrating on developing sustainable concrete by incorporating various industrial wastes such as waste glass [22], waste marble [23], silica fume [24], copper slag [25], waste foundry sand [26], cellulosic materials [27], recycled aggregate concrete [28], as well as fly ash [29,30]. In addition to utilizing different waste materials cementitious materials in concrete, RMD is also a good option.

### 1.1. RMD

**Figure 1:**
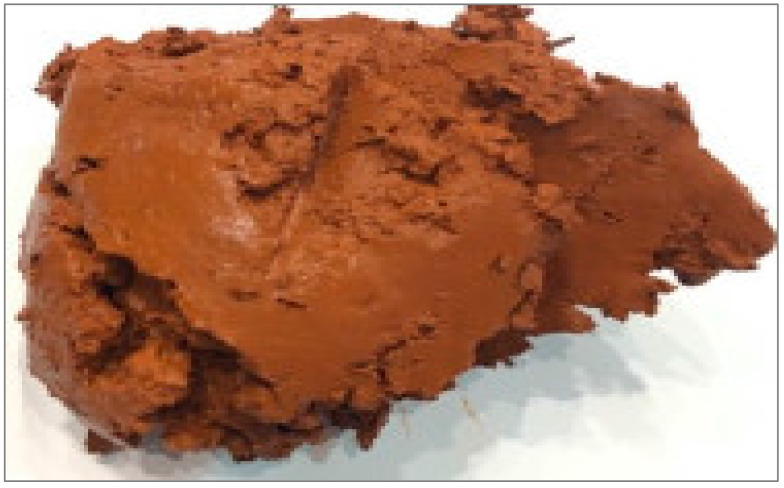
RMD [34].

Bauxite residue is an insoluble byproduct of the Bayer alumina manufacturing process. The high alkalinity of the liquid phase isolated from the RMD slurry is a major environmental concern. The challenges related to bauxite waste formation were first overlooked and ignored throughout the discovery and usage of bauxite for profit. However, when the world’s population exploded in the mid-twentieth century, resources began to deplete and waste products began to accumulate on the planet’s surface [31]. As a consequence, by the end of the twentieth century, the use of bauxite waste had become a worldwide issue. Every year, roughly 150 million tons of bauxite residue are generated worldwide [32]. In general, 1–2.5 tons of bauxite residue are created for every ton of alumina produced, with the amount varying depending on the kind of bauxite ore utilized [33]. The RMD collected from an alumina refinery is shown in Figure 1.

**Figure 2:**
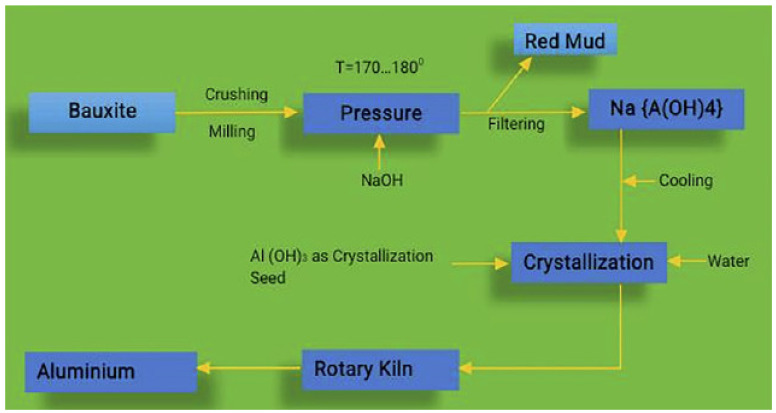
Manufacturing Process of RMD [35].

Bauxite is a mineral composed of hydrated aluminium oxides and mixtures of other elements, such as iron. The aluminium mixtures in the bauxite may be found in many kinds of aluminium parts, and contaminations may influence extraction conditions. Aluminium oxides and hydroxides are amphoteric, which means they are acidic and fundamental at the same time. Al_3_ solubility in water is minimal, although it increases dramatically with high or low PH. Bauxite metal is warmed in a weight vessel beside a sodium hydroxide setup at a temperature of 150 to 200 °C in the Bayer technique. In an extraction technique, the aluminium is broken down as sodium aluminate (basically [Al(OH)_4_]) at these temperatures. Gibbsite is accelerated when the fluid is cooled and seeded with fine-grained aluminium hydroxide precious stones from previous extractions after partitioning the accumulation by sifting. The RMD process is seen in Figure 2 [35].

**Figure 3:**
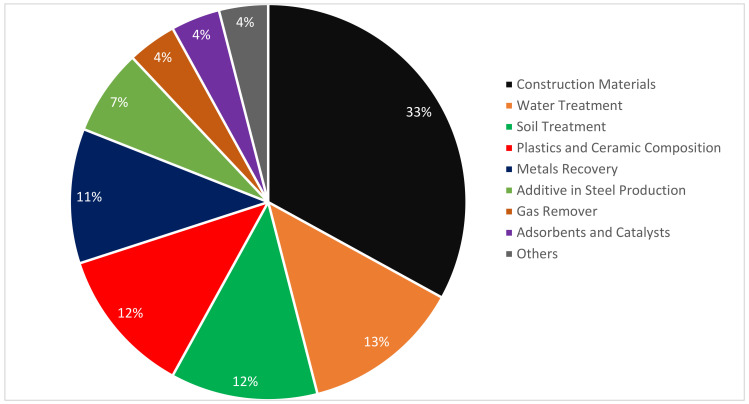
RMD Application (1964 to 2008): Data source [38].

The chemical difference in every circumstance, applying RMD is a challenge. However, detecting the RMD features for each location where it is created and establishing a template as a foundation is achievable. Two variables influence the quantity of RMD produced. The first pertains to the ore quality, while the second refers to the processing conditions. The amount of RMD produced has been approximated by many writers. The reported values vary from 0.3 to 2.5 tons per ton of poorer grade bauxite treated [36]. Patents for RMD applications were created between 1964 and 2008, according to research [37]. It is expected that 33 percent of them are used in civil building projects, as shown in Figure 3. Although RMD has a variety of uses, as seen in Figure 3, its primary use is in the building industry.

**Figure 4:**
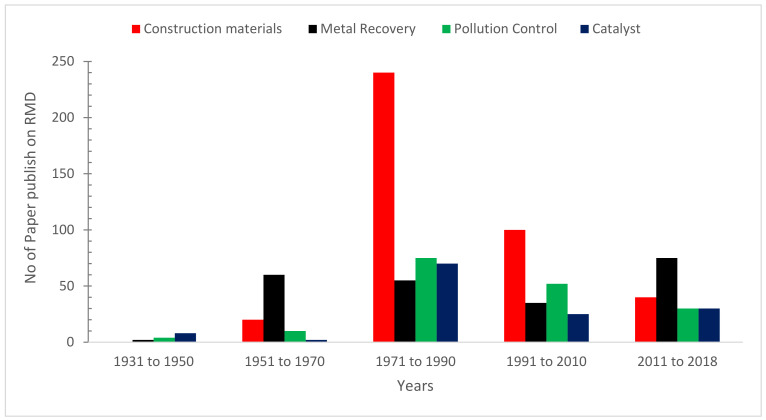
No. of Publication on RMD (1931 to 2018): Data Source [36].

Figure 4 depicts data on bauxite residue applications research publications by companies, academic institutions, and research organizations during a 20-year period. The biggest number of research articles have been published in the field of building and construction. The largest volume of RMD consultation materials was released from 1971 to 1990, and subsequently, quickly declined, as seen in Figure 4. As a result, a concise review is needed to highlight current developments and prospective applications of RDM in the construction industry.

### 1.2. Weaknesses of RMD

RMD affects agricultural landscapes due to its strong alkalinity, hence, it should be cleaned numerous times before usage. There is a chance that the RMD lake may leak. If it is organized underground, it pollutes the ground water table. Based on the writing overview, RMD fills in as an excellent folio material and has proven to be a decent cementation material. The tiny pores of cement are diminished by RMD, and as the amount of RMD in solid increments increases, the amount of water swallowed decreases. A basic combination of 70% RMD and 30% CaO yields a product with a compressive quality of 7 MPa. Ca(OH)_2,_ C_4_AH_13_ and C_4_AH_11_ are the hydrates framed after 4 days. Later investigations have confirmed these findings. When compared to fly powder, RMD does not impart much compressive quality, although flexural quality and resistance to porousness have been seen in RMD bond concrete. Despite the fact that RMD is less accessible than flies’ fiery remnants, it is critical to use or reuse RMD since it has several harmful ecological effects [39].

### 1.3. Chemical Composition

**Figure 5:**
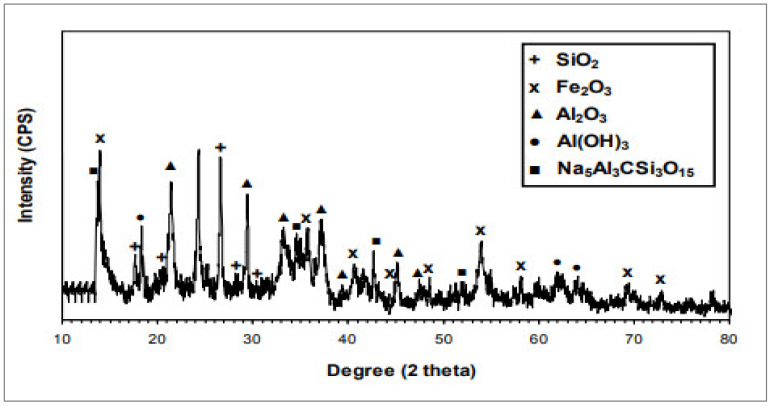
XRD of RDM [45].

Table 1 shows the chemical components of RDM while Figure 5 shows the XRD of RDM. The primary components are alumina and iron oxide, as assumed, but the relative proportions of SiO_2_ and Na_2_O are also important. In addition to aluminium hydroxide and a complicated Na_5_Al_3_CSi_3_O_15_ phase, XRD detects several of these oxides. Different researchers have reported different chemical compositions, as can be shown in Figure 5. It is likely that the chemical composition of RDM varies as the source changes. However, the accumulation of different ingredients such as silica, alumina, and iron reported by each author is higher than 50%, indicating that RDM has the potential to be employed as binding material. According to ASTM [29], the sum of the three principal oxide constituents, namely, silica, alumina, and iron, must be at least 50% for a material to be classed as pozzolanic. According to Table 1, all the RDM samples used in the various research projects may be classified as pozzolanic as per ASTM [29].

### 1.4. Significances 

The use of RMD in the manufacturing of concrete as a cement substitute has a number of environmental and economic advantages, including minimizing soil and groundwater contamination, reducing dust pollution, conserving natural resources for cement clinker, and lowering the cost of concrete production. The purpose of this paper is to present a detailed review of current progress on the use of RMD in concrete production, as well as to clearly point out four directions for using RMD in concrete production, namely, fresh properties, mechanical properties, durability aspects, and environmental aspects. Furthermore, the review also evaluates the future researcher guideline for better performance.

## 2. Fresh Properties

### 2.1. Workability

**Figure 6:**
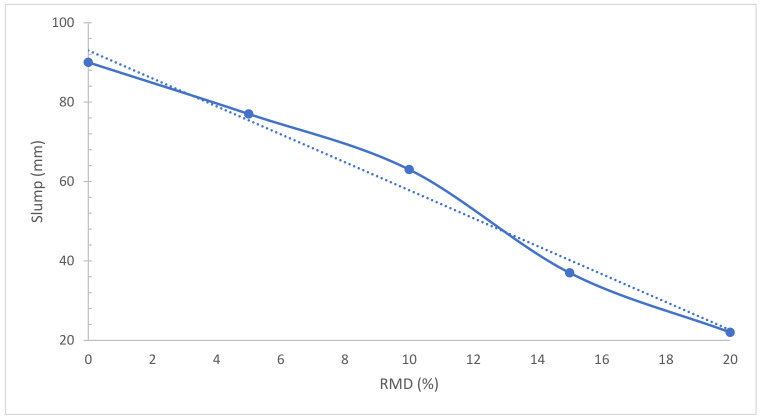
Slump Flow: Data Source [51].

As indicated in Figure 6, the slump flow diminishes dramatically as the quantity of RMD supplied boosts. The rise in adsorbed water generated by the porous nature and the greater specific surface area of RMD causes the reduction in workability. Although the addition of RMD may cause water between particles to relax, it also raises the packing density, which squeezes the free water between the particles and improves workability [46]. This might be due to the porous RMD which enhanced water absorption ability [47]. RDM, which has smaller particles than cement, reduces the fluidity of concrete by absorbing more moisture in the fresh concrete condition. However, since water made up around 48 percent of the total mass of the RDM employed in this investigation, adequate moisture could be provided in the fresh concrete phase [48]. However, the larger surface area of the seawater-neutralized bauxite refinery residence is most likely to blame for the decrease in a slump. Furthermore, unlike water added to natural sand during concrete production, water in seawater-neutralized bauxite refinery reside containing mix may not be as readily available to lubricate the mix, as it may be held within the fine-particle aggregates or chemically bound with the hygroscopic seawater-neutralized bauxite refinery reside [49]. A study [42] explored the slump flow of self-compacting concrete as per standard [50] and claimed that RMD decreased the slump flow of concrete.

### 2.2. Fresh Density

**Figure 7:**
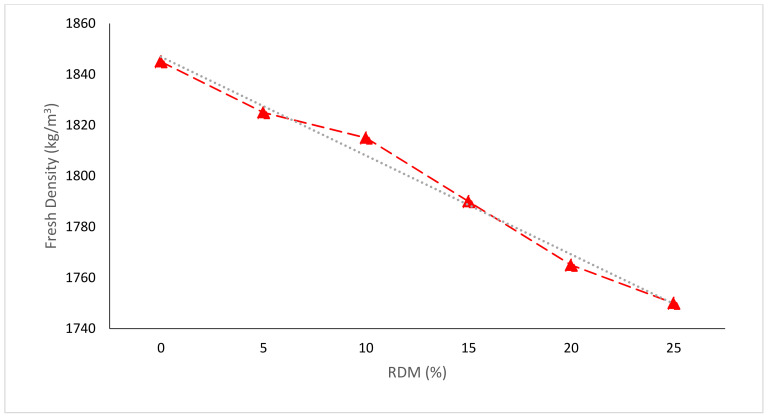
Fresh Density: Data Source [40].

Figure 7 depicts the changes in the fresh density of concrete after 28 days in relation to RDM cement replacement. The dry density ranges from 1685 to 1789 kg/m^3^, whereas the fresh density ranges from 1752 to 1844 kg/m^3^. When RDM concentration in concrete is raised, the specific gravity of the concrete drops. The specific gravity of the concrete specimen having 25% RDM is around 5% lower than the reference specimen with 0% RDM, according to the findings [40]. In contrast, a study claimed that the fresh density of concrete was enhanced due to the micro filling effect of pozzolanic material which gives more dense concrete. Furthermore, due to the pozzolanic reaction, the binding properties of concrete paste also contribute to the improved density of concrete. The combined micro filling voids and pozzolanic reaction have a positive influence on the density of concrete [52]. According to research, due to improved particle packing, there was an initial rise in density and a reduction in porosity due to micro filling voids in concrete aggregates. However, at 20% RMD addition, the behaviour becomes restrained because of additional challenges in moulding and shaping samples due to lack of flowability [45].

### 2.3. Setting Time 

**Figure 8:**
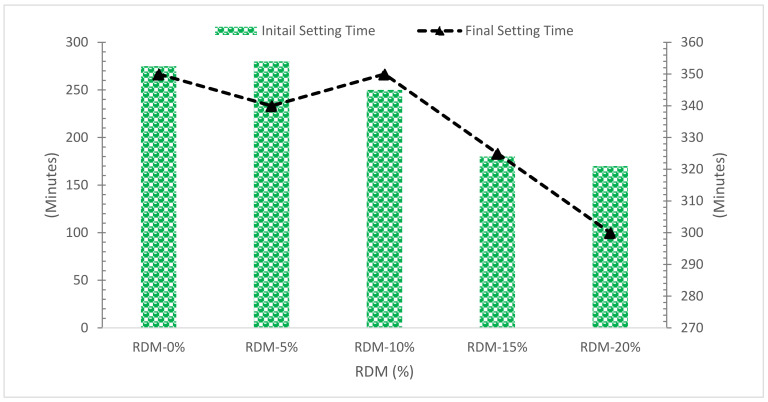
Setting Time [45].

The addition of RMD has the effect of speeding up the setting process, as shown in Figure 8. For mortars without RMD and those containing 20% waste, the end of the setting time changes from 345 to 300 min. 

The presence of aluminium and sodium hydroxides, which are known as accelerators [53], might explain this impact, in the mud, and also because of its high alkaline content. The fineness of waste particles might potentially help to retain water by competing with cement. As all formulas have the same amount of water, the leftover free fraction, which may be coupled with cement particles, will be consumed quickly [54]. In contrast, another study suggests that the pozzolanic materials decrease the setting time of cement paste as the reaction proceeds slowly [55]. The change in behavior might be possible due to a change in chemical composition.

## 3. Mechanical Properties

### 3.1. Compressive Strength (CMS)

**Figure 9:**
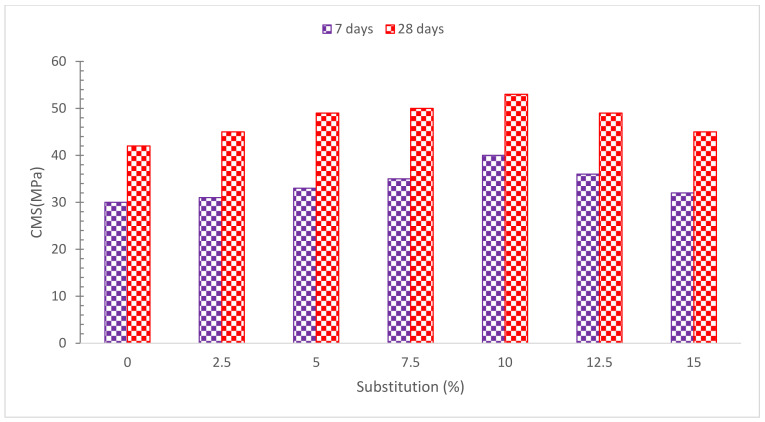
Compressive Strength: Data Source [44].

Figure 9 depicts the CMS of concrete with various RDM replacement ratios. It can be shown that with 10% RDM replacement, maximum CMS is obtained. According to research, samples with a low level of RDM had good CMS [56]. The characteristics of RMD cementitious content change when 20% of the RMD is replaced with cement by weight. As a result, more than 20% RMD substitution reduces the concrete solid mass’s compressive quality and stiffness [57]. Increases the load-bearing capability of RMD-based concrete samples by up to 10% by increasing the RMD component [58]. The self-compacting concrete (SCC) mixes created with RMD inclusion showed equivalent CMS to the control at 28 days, according to the findings. However, the impacts of red dirt content on CMS were more significant at 56 and 90 days. At 56 days, the strength of the SCC mixture samples containing 30% and 40% RMD was 89.4 MPa and 90.1 MPa, respectively, which were 8% and 9% higher than the control sample [43]. The addition of RMD had no effect on the hydration process, but when the RMD content was more than 20% (by weight of cement), the hydration of cement paste was reduced [54]. The result shows that the CMS of 20 percent partly substituted RMD concrete is higher than that of conventional concrete. This is due to an increase in the cement’s pressure quality and bond with the aggregate. As the contribution of bonding between cement and aggregate is reduced as compared to the former, increasing the amount of RMD in concrete does not result in an increase in CMS values [59]. The biggest changes were noted in the 7-day strength improvements, with 13% disparities between the 12.5 percent and 50 percent replacement levels. The major explanation is most likely owing to a high concentration of hatrurite in the cement matrix, which will be explained later. Enhanced RMD content increased CMS somewhat at 56 days, with a maximum difference of less than 4%. As a result, it is possible that RMD concentration has no effect on compressive strength, however, RMD SCC mixes in general had greater CMS than control concrete [48]. The impact of RMD on the properties of cement mortars in terms of setting time, pozzolanic activity, and mechanical strength was explored in the research. They discovered that adding RMD sped up the setting process and lowered the pozzolan reaction and that the CMS fell as the quantity of RMD increased. It was discovered that RMD may be used to partly substitute cement in non-structural mortars and concrete [60]. The compressive strength and TS of a cement mortar, in which RDM substituted up to 50% of the cement, were observed to decline when the RDM concentration was increased [48]. With an increasing amount of replacements, RMD accelerates the curing time of concrete and lowers CMS [61]. According to research, replacing 6% of RMD in concrete boosted CMS by 6%. This might be attributed to microstructure densification, as seen by the decrease in calcium hydroxide concentration after RMD replacement [62]. The strength of the concrete was reduced after 10% RMD substitution, however, it was not lower than regular concrete mixtures. CMS was lowered at 15% and 20% RMD replacement in concrete owing to inadequate cement hydration due to the presence of increased RMD content at higher replacement levels [63]. A similar justification was given by Cheng et al. [64], the large specific surface area of RMD absorbs more water in the concrete mix, resulting in a lack of water for adequate cement hydration. Table 2 shows a summary of CMS with partial substitution of RDM.

**Figure 10:**
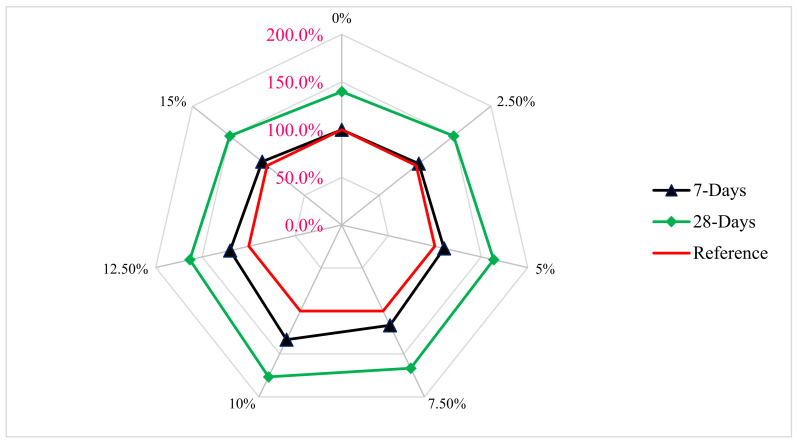
Relative Strength: Data Source [44].

Figure 10 shows a relative examination of concrete CMS with various amounts of RDM. For testing, the optimal dosage of RDM (10%) is used. The reference concrete is the control concrete’s CMS after 7 days. The CMS of RDM concrete with a 10% replacement is 33 percent higher than reference concrete after 7 days of curing. The CMS of 10% RDM replacement is 76 percent higher than reference concrete CMS after 28 days of curing. Furthermore, after 7 and 28 days of curing, all the RDM replaced mix had higher CMS than reference concrete (control concrete CMS at 7 and 28 days).

### 3.2. Flexural Strength (FLS)

**Figure 11:**
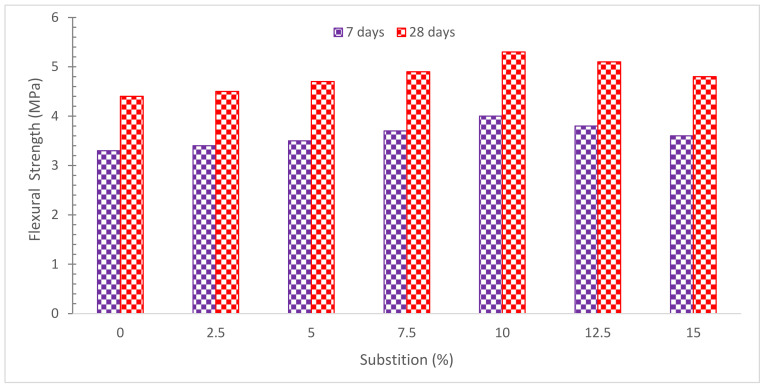
Flexural Strength: Data Source [44].

Figure 11 depicts the FLS of concrete with various RDM replacement ratios. In a similar approach to compressive strength, maximum FLS is reached at 10% RDM replacement. However, according to one study, FLS diminishes as the amount of RDM increases [40]. The FLS of concrete dropped by 1.6, 10.7, 17, 17.6 and 28.4% for RDM levels of 5, 10, 15, 20, and 25% replacement, respectively, compared to the reference sample with no RDM [40]. Tang et al. [70] found that increasing the RDM concentration as a cement substitute reduced flexural strength. According to research [71], increasing the RDM content of a hybrid composite produced using RDM as a filler and sisal fiber as the reinforcement in a polyester matrix boosts the tensile and impact strengths by up to 20%. Under tensile and impact loadings, the composite showed good resistance to fracture formation and propagation. Another study found that adding RDM to a polyester composite reinforced with sisal and banana fibers improved impact and flexural strength, making it ideal for applications requiring high load-bearing capability [72]. Ganeshan et al. [73] discovered that adding RDM to natural fiber–polyester composites significantly boosted the flexural strengths of the polyester composites while lowering the TSs. The findings of research on self-compacted concrete using a mixture of 10% iron ore and 2% RDM by cement weight indicated that the flexural, tensile, and compressive strengths were raised [74]. According to research, when RDM concentration grows, compressive and FLS falls, yet, 5% of RDM addition produces superior results [75]. A study explored using RDM as a substitute for fly ash in self-compacting mortar and concrete. RDM shows lowered the flowability of mortar and concrete but increased their compressive and flexural strengths [76]. The 28-day FLS of ultra-high-performance concrete (UHPC) with 20%, 40%, 60%, and 70% RDM (by volume and mass) is 41.6 MPa, 37.6 MPa, 35.3 MPa, 19.6 MPa, 40.7 MPa, and 32.4 MPa, respectively. Due to the toughening effect of steel fiber, the drop in FLS is not as noticeable as the decrease in compressive strength. Steel fiber toughening is the most important factor in flexural strength. As a consequence, the FLS loss caused by RMD is less noticeable than the CMS deterioration [46]. The FLS of concrete with 30% RDM substitution is greater than the other test samples [59]. Table 3 shows a summary of FLS with partial substitution of RDM. It can also be noticed that fewer studies consider FLS of concrete with RDM.

**Figure 12:**
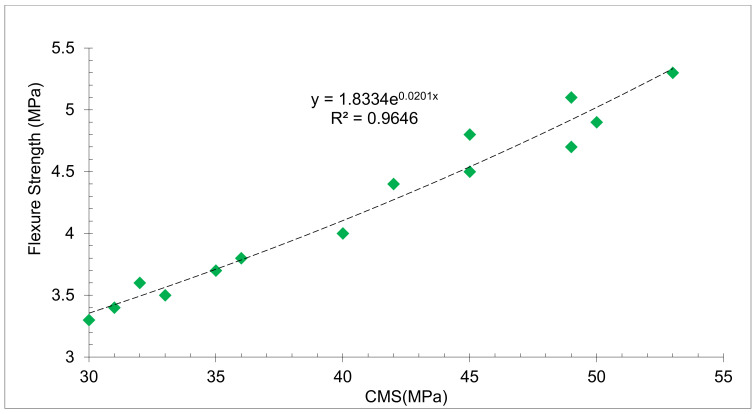
Correlation between Flexural and Compressive Strength: Data Source [44]. Green diamond is the data point.

Figure 12 depicts the relationship between compressive and FLS with various RDM replacement ratios at 7 and 28 days of curing. With an R square value larger than 0.90, a substantial connection between CMS and FLS may be noticed. As a result, the linear regression equation may be used to forecast the FLS of concrete.

### 3.3. Split Tensile Strength (TS)

**Figure 13:**
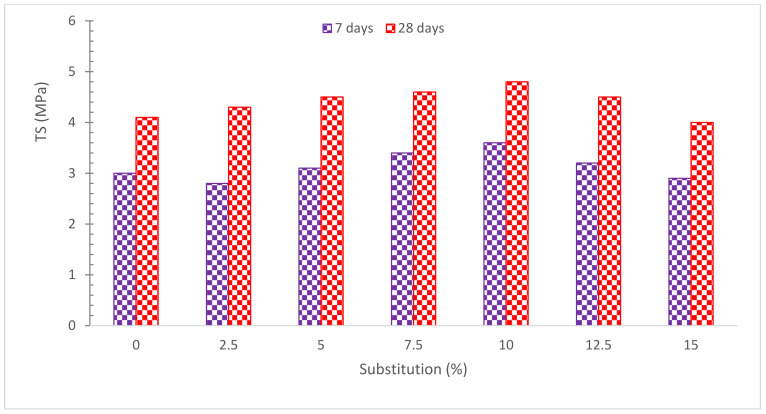
Tensile Strength: Data Source [44].

The TS of concrete with different substitution ratios of RDM is shown in Figure 13. It can be observed that maximum TS is achieved at 10% substitution of RDM in a similar way to compressive strength. According to research [63], the strength of concrete improved up to 10% substitution of RDM, and more substations of RDM after 10% caused the strength to decrease. Another study found that when up to 2.5 percent of cement was replaced with RDM, the splitting TS rose before declining as the RDM content was increased [6]. When compared to normal concrete, the strength parameters of concrete, such as compressive strength and TS, improve with a 20% substitution of RDM [59]. Increases in RDM content seem to result in minor increases in CMS and elastic modulus, as well as a little loss in TS [48]. According to research, the SCC with 2% RMD and 10% iron ore tailings had the greatest compressive, tensile and flexural strengths [77]. Furthermore, the internal curing of the RDM might be the cause of TS growth at higher curing ages [78]. There was no significant variation in TS across any of the samples, particularly after 56 days. The inclusion of RMD had no significant effect on the TS of SCC, according to the findings [48]. For 28 and 56 days, the RMD concrete had lower TS than the control, but for 90 days, the RMD concrete had higher TS than the control. The porosity of RMD was credited with increasing its tensile and compressive strength. It was said that RMD absorbed water and then released it later to help with hydration [43]. The optimal value of split TS was reached by replacing 10% of the cement with neutralized RMD and adding 5% of hydrated lime [39]. Furthermore, Table 4 shows summary of TS with partial substitution of RDM.

**Figure 14:**
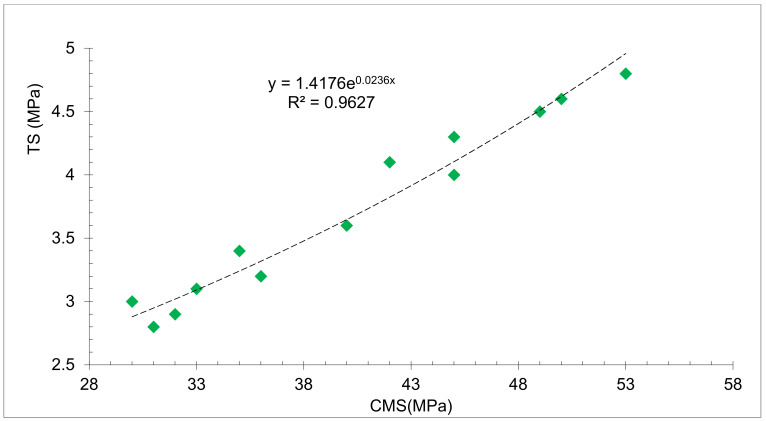
Correlation between Tensile and Compressive Strength: Data Source [44]. Green diamond is data point.

Figure 14 shows the correlation between the CMS and TS with different substitution ratios of RDM at 7 and 28 days’ curing. It can be seen that a strong correlation between CMS and TS exists with an R square value greater than 0.90. Therefore, the equation developed based on linear regression can be used to predict the TS of concrete.

## 4. Durability

### 4.1. Water Absorption

**Figure 15:**
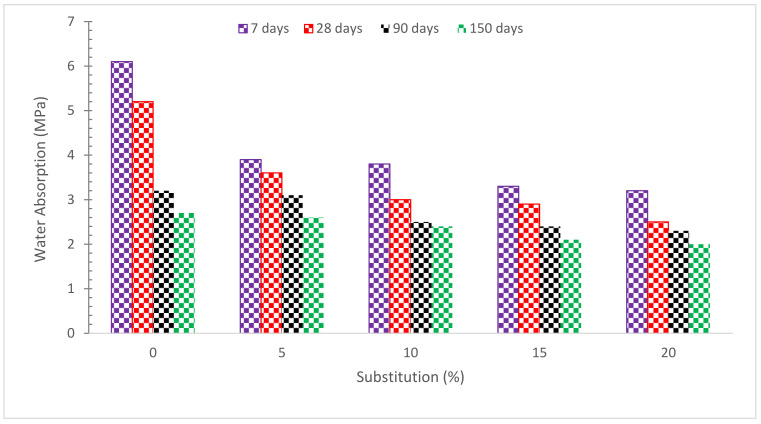
Water Absorption [63].

As indicated in Figure 15, increasing the amount of RDM replacement reduces the percentage of water absorption. The water absorption test was performed after a certain curing age, such as 7, 28, 90, and 150 days. The goal is to determine the water absorption resistance of RMD concrete as hydration progresses. The findings show that when the curing age increases and the replacement amount of RDM increases, the water absorption values decrease. The micro filling effect of pozzolanic material which gives more dense concrete by filling voids results in decreases in water absorption. Furthermore, due to the pozzolanic reaction, the binding properties of concrete paste also contribute to the improved density of concrete leading to lower water absorption. The combined micro filling voids and pozzolanic reaction have a positive influence on the water absorption of concrete [52]. RMD promotes pozzolanic activity at a later age, resulting in fewer connections between pores. The fineness of RMD particles (average particle size 14 microns) is another cause of less water absorption; all micro-cracks and holes in the concrete are filled. As a result of its enormous specific surface area, RMD may help concrete absorb less water [58]. The greater Ca(OH)_2_ crystals were fractured into multiple tiny crystals and less orientated in the RMD-based cement hydration process, which leads to minimizing pore connections and water absorption, according to Manfroi et al. 2014 [79]. Due to the existence of multiple “pits” and “folds” on the surface of the RM particles, increasing the RDM concentration had a negative influence on the water absorption of SCC, and therefore the capacity of absorbing water increased [43]. Overall, it can be claimed that as the RDM ratio grew, the ability of concrete to absorb water improved. A concrete containing 2.5 percent RDM, on the other hand, behaved similarly to the control mix [6]. The results revealed that using 15, 20, and 25% RDM as a cement substitution increased water absorption by 23 and 30%, respectively, as compared to a control specimen with no RDM. According to other research, the key explanation for the enhanced water absorption is the increased porosity caused by the usage of RDM [60,80] in terms of quality, it divides concrete into three categories: bad, average, and excellent, with water absorption of 0–3 percent, 3–5 percent, and 5% or more, respectively. Water absorption levels of over 3% and below 5% were found in all concrete specimens evaluated in this study, putting the concrete in the average absorption category. According to another study [81], high-quality concrete has a water absorption rate of less than 5%.

### 4.2. Chloride Permeability

**Figure 16:**
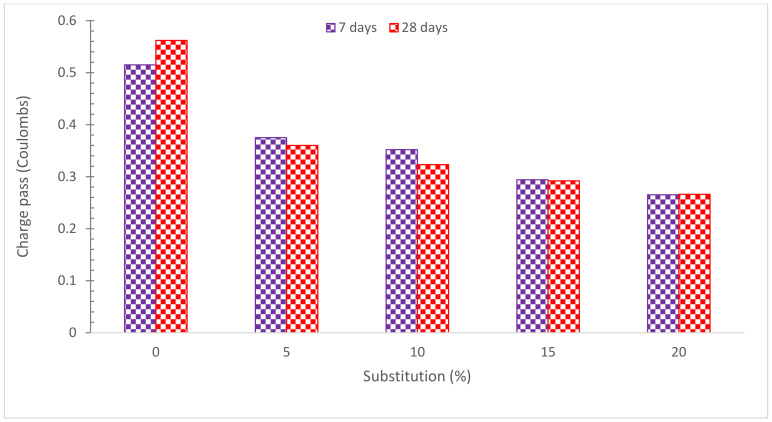
Chloride Permeability [67].

The resistance of the samples to chloride ion penetration was determined by testing the chloride permeability of the RDM-based concrete. The RCPT was performed in accordance with ASTM C 1202 [82] and the charge that travelled through the samples was measured in coulombs. Figure 16 shows the total charges that travelled through concrete samples made RDM and cured for 28 and 56 days, respectively. With increasing curing age, the resistance to chloride-ion penetration definitely increased. The reason for this is that as curing age increases, hydration products develop. The RDM-based concrete had superior chloride-ion penetration resistance than the control samples. As RDM is alkaline, it increased the resistance of the concrete to chloride ion penetration and hence reduced the total charge transferred. Thus, substituting RDM for cement in the concrete reduced the charge transmitted, indicating increased resistance to chloride ion penetration. Research indicated that tiny RM particles are responsible for the decrease of chloride ions penetration and carbonation depth [83]. The combined pozzolanic and filling voids, RDM improved the resistance of concrete against chloride-ion penetration.

### 4.3. Sorptivity Test

**Figure 17:**
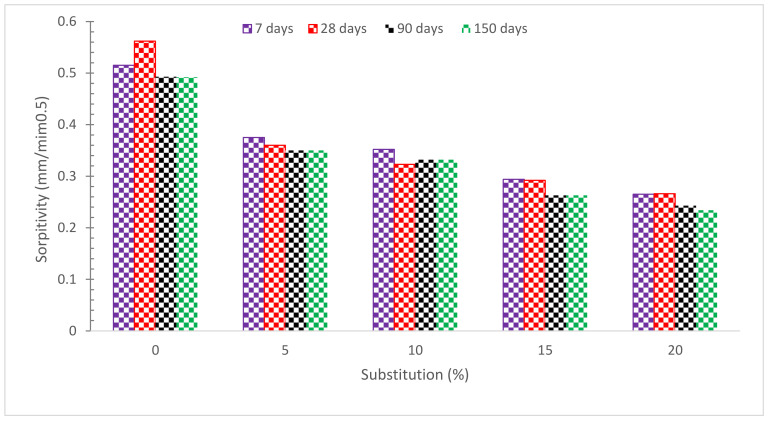
Sorptivity of Concrete [63].

With an increase in the degree of RMD replacements in concrete, the sorptivity values displayed in Figure 17 decreased. The sorptivity values are lowered after 28 days of curing from 0.562 mm/min^0.5^ (control) to 0.266 mm/min^0.5^ (20% RDM) which is ascribed to the fineness of RDM, which makes the concrete surface extremely thick by filling all the spaces. This filling property would aid in the development of a continuous pozzolanic reaction between RMD and cement, improving the strength and durability of concrete over time. As C–S–H filled all of the capillary holes during hydration, microstructure analysis indicated that replacing RMD in concrete lowered sorptivity values. This occurs because RMD fills holes and cracks in concrete, which results in lower sorptivity [83].

### 4.4. Surface Resistivity Test

**Figure 18:**
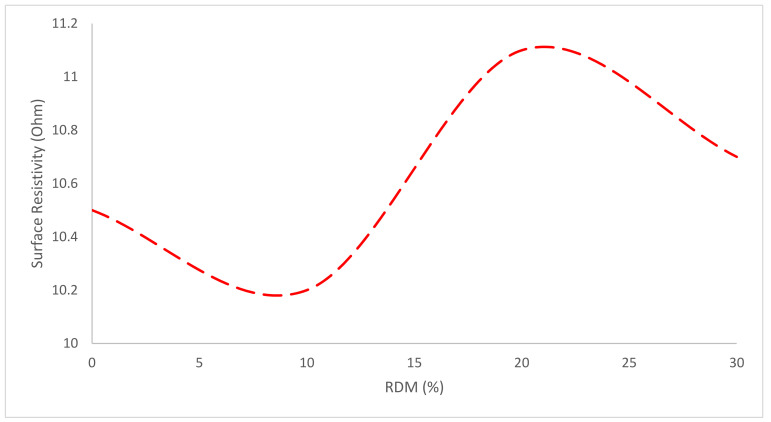
Surface Resistivity [59].

The surface resistivity test may be performed to determine the electrical resistivity of water-saturated concrete and provide a quick indication of its resistance to chloride ion penetration. The resistance to current leakage along the surface of insulating material is characterized by the substance’s surface resistivity. A voltage is transmitted between the two electrodes and a test specimen is placed between them. A resistivity meter is used to determine the value [84]. The surface resistivity of RMD-replaced concrete specimens is similar to that of normal concrete, according to test findings. The test specimens’ resistivity values are shown in Figure 18. Less information is available on the surface resistivity of concrete with RDM, and detailed investigation is required.

## 5. Microstructure Analysis

### 5.1. Interfacial Transition Zone

**Figure 19:**
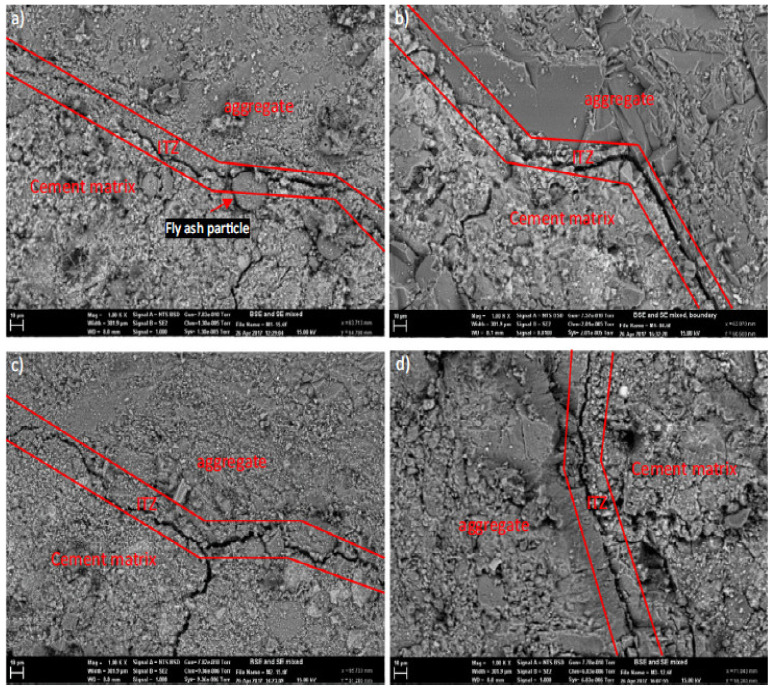
Interfacial Transition Zone (ITZ): As per Elsevier Permission [48]. (**a**) 0% (**b**) 12.5% (**c**) 25% and (**d**) 50% RMD.

The area between aggregate and paste is known as the interfacial transition zone (ITZ). When a porous fracture separates the two locations, the ITZ is at its weakest. The ITZ is highly established and must correspond to strong strength if there is just a little fracture evident or it virtually seems like one uniform surface. Figure 19 shows SEM images of ITZ of reference and RMD concrete. The dark fissures in (0% RDM) ITZ were used to identify the porosity. As demonstrated in Figure 19a, the fly ash did not bind effectively with the cement paste or aggregate, resulting in a loss in compressive and TS. RDM 12.5%, RDM 25%, and RDM 50% seem to have the same porosity ITZ as the control. The cracks of RDM 12.5%, RDM 25%, and RDM 50% were comparable in breadth to the controlled crack. In terms of porosity and microfractures, RMD concrete samples bonded similarly to control concrete samples. It can be seen from a comparison of the SEM pictures of the different mixes that there was no significant variation in ITZ for all mixes in terms of penetrability and fracture size. All blends may have identical compressive and TS [48].

When cement is hydrated, quartz and calcium oxide react, assisting in the development of CSH gel. The presence of larnite and hatrurite in the red mud concrete provides conclusive proof that CSH gel was formed, giving the concrete strength qualities. According to research [63], the Ca/Si ratios are 1.13, 0.99, 0.95, 1.01, and 1.08 for red mud replacement levels of 0%, 5%, 10%, 15%, and 20%, respectively. Based on the findings, concrete with 10% red mud replacement has a lower Ca/Si ratio than other mixtures. The creation of CSH gels increases with decreasing Ca/Si ratios and decreases with increasing Ca/Si ratios [67], which accounts for the increased strength shown in mixes with 10% red mud replacement. The findings demonstrate that RMD has a good pozzolanic activity that is comparable to that of fly ash [43].

### 5.2. Energy Dispersive Spectroscopy (EDS)

**Figure 20:**
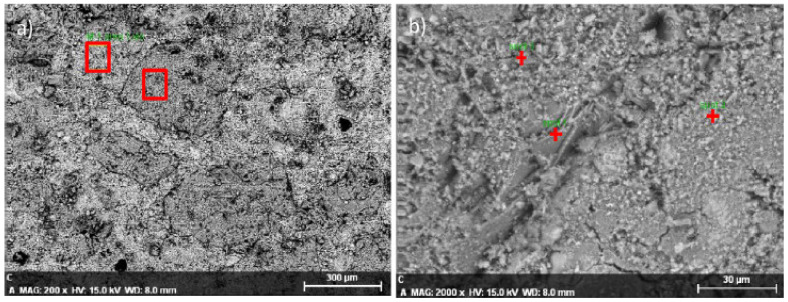
EDS Results: As per Elsevier Permission [48]. (**a**) areas (**b**) spots.

EDS was employed on the hardened cement paste samples during the SEM investigation. This would allow researchers to establish what compounds appear and whether the structure is modified significantly when RMD concentration increased compared to the control. Since calcium hydroxide intensity impacts the Ca/Si ratio of the CSH and the creation of the CSH network, the calcium/silicon (Ca/Si) ratio was tested to see how effectively the cement had formed [85]. CSH is critical for the development of strength as it works as a binder [86]. According to research, there was no significant variance in ITZ for all mix in terms of penetrability and fracture size when comparing SEM images of the various mixtures. As a result, the compressive and TSs of all mixtures may be similar [48]. The sites of the EDS analyses on the RMC0 simple are displayed in Figure 20. Table 5 reveals the quantitative findings of the elemental. The EDS data show that there was a high concentration of calcium and silicate, with a Ca/Si ratio of 1.44.C-S-H values for concrete was typically about 1.7, with CH being somewhat higher [87].

## 6. Environmental Impact Analysis

**Figure 21:**
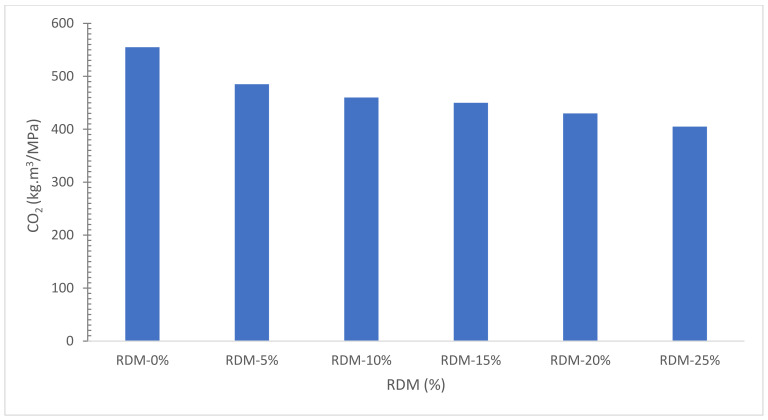
GWP during Concrete Production: Data Source [40].

Figure 21 depicts the effects of CO_2_ vs. RDM replacement in concrete. As can be shown in Figure 21, increasing the RDM concentration from 0% to 25% reduces CO_2_ from 556.8 to 409.9 kg·m^3^. As a result, CO_2_ rises in proportion to the amount of cement. In general, it can be said that cement is the source of the most CO_2_ releases. As a result, even when delivery distances are much larger than the cement delivery distance, the usage of RDM is viable.

**Figure 22:**
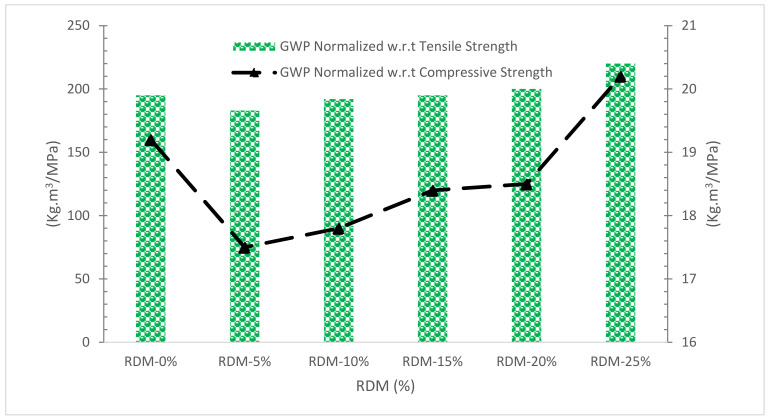
Normalized GWP W.r.t Compressive strength and TS: Data Source [40].

The concentration of CO_2_ discharges per unit volume of concrete per 28-day strength characteristics (compressive and tensile) for the concrete employed in the research are displayed in Figure 22. As this index allows for the consideration of both performance (strength) and contribution of concrete to GWP per unit volume and strength, it is characterized as a good alternative for assessing the various effects of concrete usage [88]. As can be observed, the concentration of CO_2_ discharges normalized by various strength qualities including RDM up to 20% substitute is less than the intensity of CO_2_ emissions for a reference specimen with no RDM.

**Figure 23:**
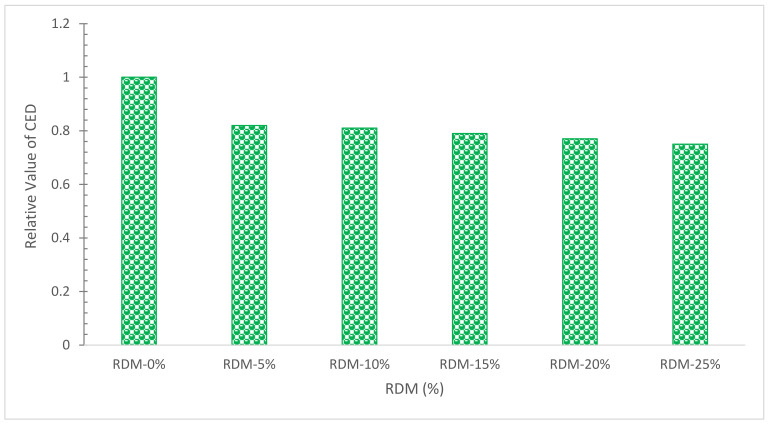
CED for Concrete with various percentages of RDM: Data Source [40].

The energy needed throughout the life cycle of lightweight concrete is shown by CED in this research. Figure 23 shows a graphical evaluation of normalized CED in concrete with various RDM contents. As can be observed, improving the RDM from 0 to 1% reduces the value of CED by around 31%.

**Figure 24:**
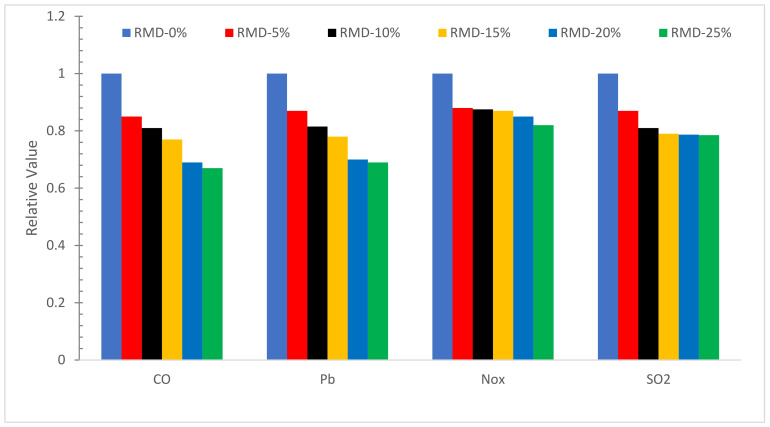
Air Pollution for Concrete with various percentages of RDM: Data Source [40].

The comparison of normalized main criterion air pollutants in concrete with varying RDM concentrations is displayed in Figure 24. It can be examined, that as the RDM content boosts, the amounts of CO, NO_X_, Pb, and SO_2_ decreased by roughly 32.5 percent, 17.1 percent, 31.8 percent, and 22.4 percent, respectively, as compared to 0 percent RDM. In general, the primary criterion air pollutants of CO, NO_X_, Pb, and SO_2_ rise with increased cement concentration owing to fuel-burning during the pyro processing step.

## 7. Conclusions

The use of RMD as a cement substitute provides a variety of environmental and economic benefits, including reduced soil and groundwater contamination, reduced dust pollution, conservation of natural resources for cement clinker, and lower concrete construction costs. The goal of this analysis is to provide a comprehensive overview of current progress in the use of RMD in concrete production, as well as to clearly identify four directions for using RMD in concrete production: fresh properties, mechanical properties, durability aspects, and environmental aspects. The detailed conclusion is given below.

- The chemical composition of RDM shows that it can be used as pozzolanic material.
- The flowability of concrete decreased as the substitution ratio of RDM increased due to its porous nature.
- Mechanical performance such as compressive strength, flexural strength and TS improved with the substation of RDM up to a certain level. Maximum CMS was achieved at 10% substitution of RDM which is 43% higher than reference concrete compressive strength. Further, the substitution of RDM results in the decreased mechanical performance of concrete. Therefore, finding an optimum is important for maximum performance. Different researchers recommend a different optimum dose of RDM. This might be possible due to different sources of RDM. However, the majority of researchers recommend 10 to 15% substitution of RDM as an optimum dose.
- Water absorption and chloride permeability decreased concrete considerably with substitution RDM. However, less information is available in this regard.
- The velocity of ultrasonic waves is reduced when the RDM concentration is increased.
- SEM results show that the substation of RDM improved the interfacial transition zone (ITZ). It is a result of the micro filling effect of RDM which fills the crack (ITZ), leading to more dense concrete.
- EDX results ensure the pozzolanic activity, creating additional compounds (CSH), which enhanced the cementitious properties of the paste.
- CO_2_, a rate of worldwide warming, reduces from 556.8 to 409.9 Kg·m^3^ as RDM content rises from 0% to 25%, showing that concrete sustainability improves as RDM content increases.
- When the RMD content is increased from 0% to 25%, the amounts of CED, CO, NO_X_, Pb, and SO_2_ are reduced by around 31%, 32.5 percent, 31.8 percent, 17.1 percent, and 22.4 percent, respectively.

Overall, the analysis reveals that RDM has the potential to be used as a cement substitute. Decreased soil and groundwater contamination, reduced dust pollution, conservation of ecological assets, and cheaper concrete production costs are some of the environmental and economic advantages of using RMD as a cement alternative in concrete manufacturing.

## 8. Recommendation

- Fewer data are accessible on durability aspects particularly dry shrinkage and creeps. Therefore, this review recommends a detailed investigation of dry shrinkage and creep properties of concrete with RDM.
- Different methods, such as thermal activation or alkali activation, should be applied to improve the pozzolanic activity of RDM.
- Thermal conductivity and heat insulation characteristics with RMD should be investigated in detail.
- Although RDM improved the strength of concrete, but concrete is still weak in tension. Therefore, this review also recommends fibers in RDM-based concrete to enhance the tensile capacity.

## Figures and Tables

**Table 1:** Chemical Composition of RMD.

Authors	[40]	[41]	[42]	[43]	[44]
SiO_2_	14.7	9.0	14.88	45.76	17.60
Al_2_O_3_	17.7	12.0	23.53	40.69	43.43
Fe_2_O_3_	27.6	37	36.48	2.85	0.65
MgO	1.7	-	1.61	0	-
CaO	14.7	6.0	1.83	4.98	2.87
Na_2_O	5.4	5.0	9.41	0	10.55
K_2_O	0.1	-	-	0.45	2.0

**Table 2:** Summary of Compressive strength.

Reference	RDM Replacement with Cement	Compression Strength (MPa)
[6]	0%, 2.5%, 5%, 7.5%, and 10%	28 Days: 42, 45, 43, 35, and 30. 56 Days: 45, 47, 45, 40, and 33.
[40]	0%, 5%, 10%, 15%, 20%, and 25%	7 Days: 21, 21, 20, 19, 18, and 17. 28 Days: 28, 27, 26, 25, 22, and 17.
[65]	0%, 10%, 20%, 30%, 40%, 50%, and 60%	28 Days: 62, 40, 110, 92, 78, 50, and 60.
[43]	0%, 10%, 20%, 30%, and 40%	28 Days: 80, 81, 81, 82, and 83. 56 Days: 82, 84, 85, 90, and 90. 90 Days: 90, 91, 92, 100, and 97.
[66]	0%, 1.0 F% + 10 B%, 1.0 F% + 20 B%, and 1.0 F% + 30 B%	7 Days: 26.95, 26, 26.35, and 25.87. 28 Days: 38.87, 35.72, 36.87, and 36. 90 Days: 47.51, 46.21, 46.58, and 44.48.
[44]	0%, 2.5%, 5%, 7.5%, 10%, 12.5%, and 15%	7 Days: 30, 31, 33, 35, 40, 36, and 32. 28 Days: 42, 45, 49, 50, 53, 49, and 45.
[34]	0%, 20%, and 40%	28 Days: 35, 29, and 18. 56 Days: 38, 32, and 18. 90 Days: 41, 35, and 19.
[51]	0%, 5%, 10%, 15%, and 20%	56 Days: 48, 48, 55, 46, and 45. 180 Days: 37, 33, 41, 41, 26, and 28.
[58]	0%, 2.5%, 5%, 7.5%, 10%, 12.5%, and 15%	UTRM: 40, 40, 43, 40, 35, 34, and 32. TRM: 40, 42, 43, 41, 45, 43, and 40.
[59]	0%, 10%, 20%, and 30%	7 Days: 20, 21, 22, and 23. 14 Days: 27, 28, 27, and 27. 28 Days: 32, 31, 35, and 33.
[46]	0%, 20%, 40%, and 60%	28 Days: 159.7, 139.8, 129.8, and 107.3
[63]	0%, 5%, 10%, 15%, and 20%	7 Days: 30.16, 33.65, 35.67, 34.54, and 31.27. 28 Days: 43.55, 45.34, 48.1, 46.05, and 44.09. 90 Days: 48.2, 55.61, 59.75, 53.79, and 52.18. 150 Days: 49.5, 61.1, 67.5, 60.2, and 57.5.
[67]	0%, 5%, 10%, 15%, and 20%	28 Days: 42, 43, 47, 44, and 41. 56 Days: 45, 46, 49, 46, and 43.
[68]	0, 96, 115, 144, and 192 kg	28 Days: 58, 77, 91, 71, and 70.
[48]	0%, 12.5%, 25%, and 50%	7 Days: 30, 35, 32, and 35. 28 Days: 47, 53, 50, and 50. 56 Days: 57, 58, 60, and 61.
[39]	0%, 10%, 20%, and 30%	7 Days: 21.09, 25.36, 24.64, and 21.53. 28 Days: 33.50, 36.47, 34.26, and 31.78.
[69]	0%, 2%, 4%, 6%, 8%, 10%, and 12%	28 Days: 40, 45, 40, 36, 34, 33, and 31.

Fiber = F; Bauxite = B; RMD = RDM; Treated RMD = UTRM; Untreated RMD = TRM.

**Table 3:** Summary of Flexural strength.

Reference	RDM Replacement with Cement	Flexure Strength (MPa)
[40]	0%, 5%, 10%, 15%, 20%, and 25%	28 Days: 8.0, 8.0, 7.1, 6.8, 6.7, and 6.0.
[65]	0%, 10%, 20%, 30%, 40%, 50%, and 60%	28 Days: 52, 30, 22, 26, 15, 13, and 24.
[44]	0%, 2.5%, 5%, 7.5%, 10%, 12.5%, and 15%	7 Days: 3.3, 3.4, 3.5, 3.7, 4.0, 3.8, and 3.6. 28 Days: 4.4, 4.5, 4.7, 4.9, 5.3, 5.1, and 4.8.
[51]	0%, 5%, 10%, 15%, and 20%	28 Days: 5.0, 5.5, 6.2, 5.8, and 6.1.
[58]	0%, 2.5%, 5%, 7.5%, 10%, 12.5%, and 15%	UTRM: 4.4, 4.3, 4.5, 4.4, 4.1, 4.0, and 3.9. TRM: 4.4, 4.4, 4.4.4.5, 4.5, 4.4, and 4.4.
[59]	0%, 10%, 20%, and 30%	28 Days: 6.2, 4.3, 6.3, and 7.0.
[46]	0%, 20%, 40%, and 60%	42.43, 41.6, 37.6, and 35.5.

**Table 4:** Summary of TS.

Reference	RDM Replacement with Cement	Split TS (MPa)
[6]	0%, 2.5%, 5%, 7.5%, and 10%	28 Days: 4.8, 5.2, 4.8, 3.9, and 3.5. 56 Days: 5.1, 5.3, 5.1, 4.3, and 3.8.
[40]	0%, 5%, 10%, 15%, 20%, and 25%	7 Days:1.8, 1.7, 1.6, 1.6, 1.6, and 1.5. 28 Days: 2.8, 2.7, 2.5, 2.3, 2.2, and 1.8.
[65]	0%, 10%, 20%, 30%, 40%, 50%, and 60%	28 Days: 30, 20, 13, 08, 10, 07, and 04.
[43]	0%, 10%, 20%, 30%, and 40%	28 Days: 5.2, 5.2, 5.0, 5.5, and 5.8. 56 Days: 5.7, 5.4, 5.2, 5.8, and 5.9. 90 Days: 6.0, 6.0, 6.0, 7.2, and 7.3.
[66]	0%, 1.0 F% + 10 B%, 1.0 F% + 20 B%, and 1.0 F% + 30 B%	28 Days: 1.73, 2.16, 2.34, and 1.99. 56 Days: 2.42, 2.76, 3.0, and 2.37. 90 Days: 2.97, 3.30, 3.59, and 2.75.
[44]	0%, 2.5%, 5%, 7.5%, 10%, 12.5%, and 15%	7 Days: 3.0, 2.8, 3.1, 3.4, 3.6, 3.2, and 2.9. 28 Days: 4.1, 4.3, 4.5, 4.6, 4.8, 4.5, and 4.0.
[58]	0%, 2.5%, 5%, 7.5%, 10%, 12.5%, and 15%	UTRM: 3.8, 4.1, 4.3, 4.1, 3.8, 3.7, and 3.7. TRM: 3.8, 4.2, 4.2, 4.2, 4.5, 4.2, and 4.1.
[59]	0%, 10%, 20%, and 30%	28 Days: 2.9, 2.5, 3.3, and 3.0.
[67]	0%, 5%, 10%, 15%, and 20%	28 Days: 4.2, 4.8, 5.0, 4.6, and 4.4. 56 Days: 4.6, 5.0, 5.2, 4.8, and 4.6.
[68]	0 kg, 96 kg, 115 kg, 144 kg, and 192 kg	28 Days:7.5, 9.5, 10.3, 9.7, and 8.7.
[48]	0%, 12.5%, 25%, and 50%	28 Days: 4.6, 4.7, 4.4, and 4.6. 56 Days: 4.8, 4.8, 5.0, and 4.9.
[39]	0%, 10%, 20%, and 30%	7 Days: 2.1, 2.6, 2.2, and 2.1. 28 Days: 2.1, 2.6, 2.2, and 2.1.

**Table 5:** Energy dispersive spectroscopy (EDS) Results [48].

Chemical Name	Control	RDM-12.5%	RDM-25%	RDM-50%
Na	0.74	1.70	1.43	5.53
Mg	0.69	0.57	0.87	1232.47
Al	4.09	12.82	4.99	13.03
Si	37.38	33.98	50.05	28.03
P	-	-	-	0.08
S	0.67	0.08	0.91	2.39
CI	-	-	-	0.28
K	0.86	3.40	1.30	1.60
Ca	53.96	42.87	38.28	37.76
Ti	-	1.51	-	0.92
Fe	1.61	3.07	2.17	8.20

## Data Availability

All the data available in main text.
